# A Small-Object-Detection Algorithm Based on LiDAR Point-Cloud Clustering for Autonomous Vehicles

**DOI:** 10.3390/s24165423

**Published:** 2024-08-22

**Authors:** Zhibing Duan, Jinju Shao, Meng Zhang, Jinlei Zhang, Zhipeng Zhai

**Affiliations:** School of Transportation and Vehicle Engineering, Shandong University of Technology, Zibo 255000, China; zhibingduan6@gmail.com (Z.D.); zhm990106@gmail.com (M.Z.); hucuijuan@jaran.com.cn (J.Z.); zhipengzhai31@gmail.com (Z.Z.)

**Keywords:** LiDAR, autonomous driving, ground segmentation, point cloud clustering, small object detection

## Abstract

3D object-detection based on LiDAR point clouds can help driverless vehicles detect obstacles. However, the existing point-cloud-based object-detection methods are generally ineffective in detecting small objects such as pedestrians and cyclists. Therefore, a small-object-detection algorithm based on clustering is proposed. Firstly, a new segmented ground-point clouds segmentation algorithm is proposed, which filters out the object point clouds according to the heuristic rules and realizes the ground segmentation by multi-region plane-fitting. Then, the small-object point cloud is clustered using an improved DBSCAN clustering algorithm. The K-means++ algorithm for pre-clustering is used, the neighborhood radius is adaptively adjusted according to the distance, and the core point search method of the original algorithm is improved. Finally, the detection of small objects is completed using the directional wraparound box model. After extensive experiments, it was shown that the precision and recall of our proposed ground-segmentation algorithm reached 91.86% and 92.70%, respectively, and the improved DBSCAN clustering algorithm improved the recall of pedestrians and cyclists by 15.89% and 9.50%, respectively. In addition, visualization experiments confirmed that our proposed small-object-detection algorithm based on the point-cloud clustering method can realize the accurate detection of small objects.

## 1. Introduction

Driverless cars [1] can reduce traffic congestion and accidents and improve the efficiency of social production and people’s lives. Driverless cars rely on sensors to sense their surroundings. Currently, the mainstream sensors used are cameras, light detection and ranging (LiDAR), and millimeter-wave radar, among which LiDAR is widely used in driverless cars because of its all-weather operation, high-precision ranging, and high-resolution map building. LiDAR can determine the distance and orientation of an object by emitting laser pulses with known angles and measuring their reflection times; however, it cannot acquire higher-level semantic information (e.g., information on size and category) about the obstacle. Consequently, three-dimensional (3D) object-detection algorithms based on LiDAR point clouds have emerged. Currently, there are two object-detection techniques based on LiDAR point clouds: object-detection algorithms based on point-cloud clustering and those based on deep learning.

Deep learning-based object-detection algorithms [2,3,4,5,6,7,8,9] have been iterated and optimized for several years and have a strong expressive ability and high detection accuracy in complex scenes. Initially, researchers focused on single-sensor detection, such as the YOLO algorithm for detecting objects in camera color images [10] and the PointRCNN algorithm for detecting objects in LiDAR point clouds, both of which made significant advancements in the field of object-detection [9]. Later, researchers discovered that combining information from multiple sensors could potentially yield better results. Gao H et al. proposed an object-detection method based on the fusion of camera and LiDAR data. Their approach involves initially extracting depth features from the point cloud data, which are then fused with the RGB data from the camera and input into a convolutional neural network. This process provides richer feature representations for the object-detection task, leading to further improvements in detection accuracy [11]. However, these methods require a large number of manually labeled datasets for training [12], which leads to poor generalizability; moreover, these algorithms must be designed with a large number of parametric quantities, which makes their reasoning process time-consuming [13] and, thus, it is difficult to meet real-time requirements. The general processes of object-detection algorithms based on point-cloud clustering [14,15,16] include point-cloud preprocessing, ground segmentation, object clustering, and enclosing frame fitting. In contrast, the advantage of object-detection methods based on point-cloud clustering lies in the simplicity of their design and their strong generalization, which is of great advantage in simple parks, suburbs, the countryside, and other specific scenarios.

**Figure 1:**
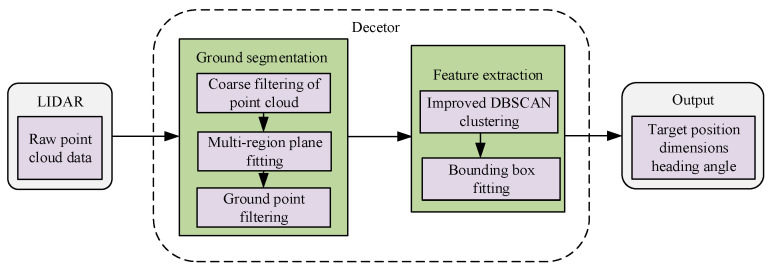
Improved small-object-detection algorithm flow based on the point-cloud clustering method.

In real-world traffic scenarios, vehicles, pedestrians, and cyclists are the most important traffic participants, as well as the most common and challenging types of targets in road environments. Their detection and recognition are crucial. A common issue with existing methods is that the detection accuracy for vehicles is significantly higher than for cyclists and pedestrians. The most apparent reason for this is the relatively smaller size of cyclists and pedestrians, making them harder to detect. Therefore, we classify cyclists and pedestrians as small targets [17]. The current object-detection algorithms based on point-cloud clustering also have low detection accuracy for small objects. The reason for this is that the existing ground-segmentation methods are prone to mistaking some small-object points for ground points. This results in a smaller number of small-object point clouds that are originally sparse, which increases the difficulty of small-object recognition and increases the difficulty of object clustering because of the large mass of point cloud data and inhomogeneity of the distribution. To solve these problems and improve the efficiency and accuracy of small-object-detection, we propose a small-object-detection algorithm based on point-cloud clustering. First, the existing algorithms have difficulty in accurately segmenting small-object points and ground points. Therefore, we propose a segmented ground-point cloud accurate segmentation algorithm that coarsely filters the object point cloud by formulating heuristic rules and completes the accurate segmentation of the ground by adopting the multi-region planar fitting method. On this basis, considering the problems of poor real-time clustering and dependence on fixed parameters of the traditional density-based spatial clustering of applications with noise (DBSCAN) algorithm [18], we adopted the K-means++ [19] algorithm for pre-clustering, use the adaptive neighborhood radius to improve the clustering effect on small objects, and improve the original algorithm’s core point search method to improve the clustering efficiency. Finally, we use the directional bounding box model to construct bounding boxes for the clustering results, in order to obtain the static information of small objects. This process is illustrated in Figure 1. In summary, our main contributions are as follows:

- A segmented ground-point cloud accurate segmentation algorithm is proposed. The object point clouds such as special points, approach points, object internal points, and departure points are filtered out according to the heuristic rules. According to the near-dense and far-sparse distributions of the point clouds, a multi-region polar coordinate meshing is adopted, and the ground segmentation is realized by plane-fitting. The proposed segmentation algorithm is qualitatively evaluated and compared with random sample consensus (RANSAC) and region-wise ground plane fitting (R-GPF). The proposed ground-segmentation algorithm can solve the problem of it being difficult to accurately segment the object point cloud and the ground-point cloud in different scenarios;
- In view of the shortcomings of the DBSCAN algorithm in terms of parameter sensitivity and poor real-time performance, an improved DBSCAN algorithm is proposed. The K-means++ algorithm is adopted for pre-clustering. The neighborhood radius is adaptively adjusted according to the distance, and the core point search method of the original algorithm is improved to reduce the number of traversal points. The improved DBSCAN algorithm can improve the clustering ability of adjacent small objects and long-distance small objects, and improve the clustering speed;
- The ground-segmentation algorithm is verified in a rough road-surface scene and a sloping road-surface scene, respectively. The clustering effect of the proposed improved DBSCAN algorithm on small-object point clouds is verified in real vehicle experiments. The experimental results showed that the improved small-object-detection algorithm based on the point cloud clustering method proposed in this paper can achieve the accurate detection of small objects.

The rest of this paper is composed as follows: Section 2 includes a literature review on ground-segmentation methods and clustered object-detection. Section 3 details our proposed algorithm for small-object-detection, including the proposed ground-segmentation method and the improved clustered-object-detection method. Section 4 presents the experiments and the analysis of the experimental results. The data used in the experiment and the settings of the experimental hardware, software, and experimental parameters are first introduced. Then, the effectiveness of our algorithm compared to other algorithms is demonstrated by control and visualization experiments in different scenarios. Section 5 contains the summarizing conclusions as well as future perspectives.

## 2. Literature Review

### 2.1. Ground Segmentation

Accurate ground segmentation can completely separate the object point cloud from the ground-point cloud, which is a prerequisite for improving the accuracy of small-object-detection. 

Ground-segmentation algorithms can be categorized based on different segmentation methods, such as elevation-based methods, slope-based methods, and ground model-based methods.

Elevation-based ground-segmentation methods primarily extract the height values of point clouds, treating lower points as ground points. This approach is simple and easy to implement, but using a single height threshold can easily lead to misclassification. Qu, W. proposed a ground-segmentation algorithm that considers multiple features, using height and related features to evaluate point clouds in a grid, improving the algorithm’s performance on unstructured roads. However, the need for multiple judgment processes reduces its real-time performance [20]. A ground-segmentation algorithm based on height maps proposed by Arora et al. [21] stores the point cloud height information in a divided cell grid. By analyzing the height features in each subgrid, the ground can be more accurately identified in a local area; however, owing to the inability to capture large height variations, sloping ground can be poorly detected. 

For this reason, Cheng et al. proposed a ground-segmentation algorithm based on scanning line segment features [22] which first calculates the height differences between adjacent scanning line segments, then introduces slope features to validate and update the results, and then classifies line segments within the threshold range as sloped ground. This method can effectively recognize sloped ground; however, the selection of the slope threshold has a significant impact on the segmentation effect. The slope-based methods involve adaptive slope thresholding for 3D point cloud ground segmentation. Zuo et al. projected the 3D point cloud data into a sectoral grid and sorted the points by their distance from the LiDAR. They segmented the points in the section based on their height and the angular relationships between adjacent points, updating the ground height and angular thresholds between the adjacent points and the segmented ground points. This method achieves good segmentation results on complex terrains but tends to be overly focused on point-to-point relationships, making it susceptible to local noise interference [23].

To ensure global consistency, ground model-based segmentation methods have emerged. These methods construct mathematical models of ground shapes and select ground seed points, using plane-fitting methods to accurately identify and segment the ground. In recent years, some scholars have conducted in-depth research on optimizing ground-model construction. Yan, Y. et al. divided the regular grid to build their ground model and employed a two-stage fitting process to improve the ground segmentation in complex scenes. However, the algorithm’s accuracy is sensitive to grid-size and slope thresholds, resulting in poor adaptability and low efficiency [24]. Accordingly, Lim et al. proposed the R-GPF method [25], which uses a polar coordinate raster to construct the ground model and plane-fitting using a principal component analysis algorithm that can effectively segment complex ground in different scenes. However, this method uses height features in the raster for ground filtering, making it easy to filter out parts of the point cloud where small objects are closer to the ground.

To solve the above problems, our proposed method filters out the object point clouds using heuristic rules to eliminate the object point cloud interference in the ground fitting process. Simultaneously, the multi-area polar coordinate grid division method is adopted to adapt to the characteristics of LiDAR point clouds, which are dense nearby and sparse far away, to prevent situations in which the number of point clouds near the ground is excessive and the number of point clouds far away is too small.

### 2.2. Point-Cloud Clustering

Point cloud clustering-based object-detection algorithms can be categorized by their clustering methods, such as depth map-based clustering, grid-based clustering, Euclidean clustering, and density-based clustering. 

The depth-map-based clustering method [26] projects the point cloud data onto a two-dimensional (2D) depth map and realizes clustering detection by processing the depth image. Although this method simplifies processing, some 3D information is lost during the projection process, and the accuracy and reliability of the depth information are affected by the presence of noise in the point cloud data. Fang, B. et al. employed denoising and restoration techniques on depth maps to improve the authenticity of the depth information, followed by clustering using the K-means clustering algorithm (K-means). However, the limited pixel resolution of depth maps impacts the detection accuracy of highly dynamic objects to some extent [27].

The grid-based clustering method [28] constructs an elevation map by dividing the 3D point cloud data into multiple 2D grids and clustering the subgrids with objects according to their features, which is more accurate than depth map clustering; however, it requires the storage of a large amount of point cloud data, which leads to the high consumption of computational resources.

The density-based clustering method uses the density features of a point cloud to cluster point clouds of different shapes and uneven densities. Currently, significant research on the density-based spatial clustering of applications with noise (DBSCAN) focuses on reducing the algorithm complexity and selecting optimal parameters. Sun et al. divided non-ground-point cloud data into small cubes using a data cube partitioning method. If the number of points in a cube exceeds the density threshold, all data points in that cube are marked as core points, reducing the excessive memory usage and improving the real-time performance of the clustering. They also set a neighborhood radius every 10 m, enhancing the point cloud clustering accuracy at different distances [29]. However, selection of the optimal threshold requires many experiments and has poor applicability. Zhou et al. employed the chameleon swarm algorithm (CSA) for adaptive searching of the neighborhood radius parameter in the DBSCAN clustering algorithm, iteratively optimizing to obtain the best neighborhood radius parameter, reducing the parameter adjustment complexity and addressing the sensitivity of the neighborhood radius to clustering results. However, manual setting of the density threshold is still required [30].

Falahiazar Z et al. proposed a hybrid algorithm using a multi-objective genetic algorithm (MOGA) to automatically determine DBSCAN algorithm parameters. The MOGA-DBSCAN algorithm views the target point cloud clustering as a multi-objective optimization problem, optimizing clustering validity indices to evaluate the quality of clustering solutions. This approach allows for adaptive adjustment of the DBSCAN neighborhood radius and density threshold parameters but has issues with clustering accuracy for dynamic targets [31].

Euclidean distance-based clustering is a method that calculates the Euclidean distance between points in the point cloud data through continuous iteration, and groups the points with distances less than a preset threshold into one class, thus realizing object-detection. Sun et al. proposed a multi-threshold Euclidean clustering method [32] which obtains a better clustering effect by setting multiple thresholds at different distances; however, this method still needs the manual setting of thresholds, which restricts its clustering performance in different scenarios. Qu et al. determined an adaptive distance threshold based on factors such as the LiDAR horizontal and vertical resolutions, adjustment functions, and tuning parameters. They employed a 3D k-dimension tree to establish search relationships among discrete points, maintaining the high real-time performance of traditional Euclidean clustering methods while improving the clustering accuracy for sparse distant target point clouds. However, the optimal adaptive distance threshold still requires extensive experimentation for adjustment [33].

In summary, the method based on Euclidean distance clustering is simple, intuitive, and efficient but suffers from the problems of parameter sensitivity and poor real-time performance. Our improved algorithm solves the problem of a poor clustering effect due to parameter sensitivity by setting the adaptive neighborhood radius and improves the clustering speed by improving the searching of core points.

## 3. Methods

### 3.1. Ground-Point Cloud Segmentation

**Figure 2:**
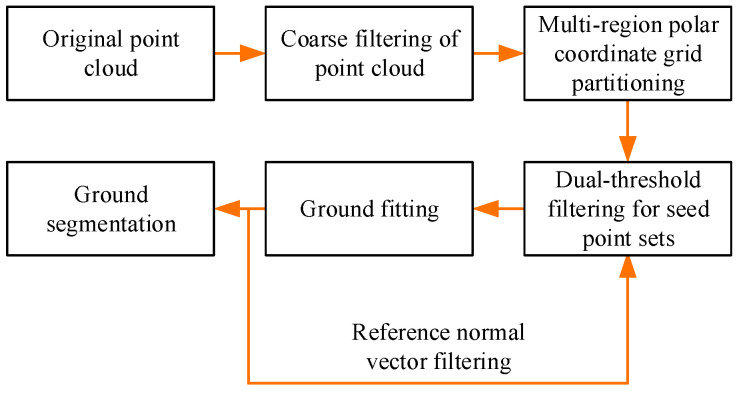
Segmented ground-point cloud accurate segmentation algorithm flow chart.

To improve the ground-segmentation effect while considering real-time performance, we propose a segmented ground-point cloud accurate segmentation algorithm, as shown in the block diagram in Figure 2. In the first stage, according to the distribution characteristics of the small-object point cloud, the preprocessed object point cloud is coarsely filtered based on the heuristic rule. In the second stage, the near (dense) and far (sparse) physical characteristics of the 3D point cloud are adjusted, and a multi-region polar coordinate grid model is constructed for the 3D space. Simultaneously, to consider real time, the seed-point set is screened by using a double threshold in a fast and efficient way. Finally, the relationships between subgrids and surrounding neighboring subgrids are considered in fitting the whole plane, and a reference normal is introduced to the subgrids. Finally, the relationship between the subgrid and the surrounding neighboring subgrids is considered in fitting the entire plane, reference normal vectors are introduced to further validate the fitting results, and the entire ground-point cloud is segmented.

#### 3.1.1. Coarse Filtering of Point Clouds

LiDAR scanning produces very few point clouds on the surfaces of cyclists and pedestrians compared to those of vehicle objects. In this case, points with lower height values in the small-object point cloud are easily misclassified as ground points, affecting the accurate fitting of the surface plane. Therefore, it is necessary to filter out most small-object point clouds during ground segmentation.

For this reason, we propose a heuristic rule to filter most of the object points. The design of the heuristic rule is mainly based on the special geometric relationship between object points and ground points in the point cloud data. Specifically, the heuristic rule first determines whether a point is a special point by the angle threshold between the points and the distance between the points and the sensor. Then, the height threshold is used to judge whether the special point is an object point, in order to achieve the purpose of filtering the object points. At the same time, in order to adapt to the distribution characteristics of the LiDAR point cloud near-dense and far-sparse points, an adaptive threshold parameter is used in setting the height threshold, and its value increases with an increase in the distance, which is convenient in achieving a better screening effect.

**Figure 3:**
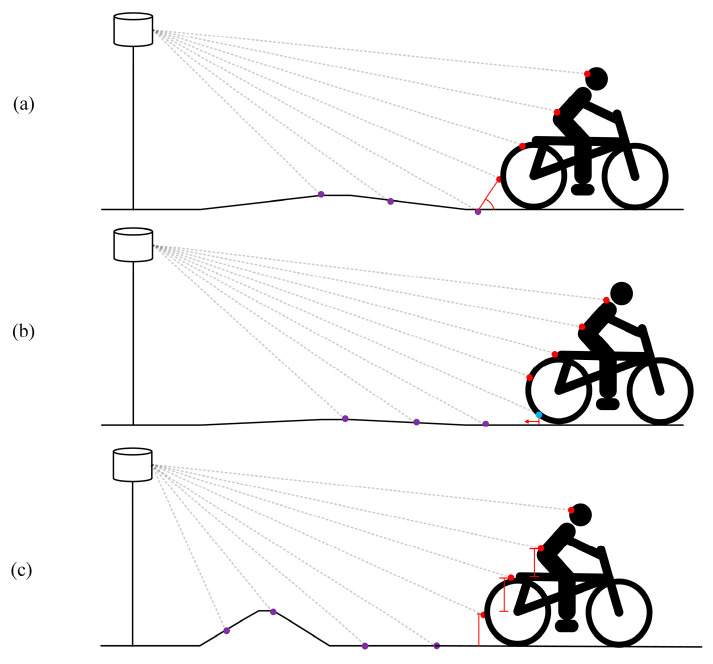
Example of object and ground classification. (**a**–**c**), respectively, describe the situations of object points and ground points under different road conditions.

At the same horizontal azimuth, the point cloud is sorted according to the vertical angle of the laser beam, and the relationship between these points is analyzed from near to far. Four types of point cloud labels can be obtained: special point, approach point, object internal point, and departure point. Based on the geometric distribution of the point cloud, different heuristic filtering rules were defined and represented by a cyclist, as shown in Figure 3.

(1) Special point filtering rules

For a set of ordered point sets $P_{b}$ consisting of different laser beams in the same horizontal orientation, denoted as $P_{b}=\left\{\left(x_{1},y_{1},z_{1}\right),\left(x_{2},y_{2},z_{2}\right),\ldots \ldots ,\left(x_{n},y_{n},z_{n}\right)\right\}$, two heuristic rules are defined to find special points, and satisfying one of them classifies the current point as a special point.

Rule 1: If the angle between the previous point $p_{\left(i-1\right)}=\left(x_{\left(i-1\right)},y_{\left(i-1\right)},z_{\left(i-1\right)}\right)$ and the current point $p_{i}=\left(x_{i},y_{i},z_{i}\right)$ exceeds the threshold $\alpha_{th}$, the current point $p_{i}$ is considered a special point:

(1) $$arctan\left(\frac{z_{i}-z_{\left(i-1\right)}}{\sqrt{{\left(x_{i}-x_{\left(i-1\right)}\right)}^{2}+{\left(y_{i}-y_{\left(i-1\right)}\right)}^{2}}}\right)>\alpha_{th}$$

There are two possibilities for the points identified based on Rule 1: one is that it is a small-object point, as shown in Figure 3a; at the same time, it may be a ground point with a large change in slope, as shown in Figure 3c. Therefore, the points identified based on Rule 1 are categorized as special points only.

Rule 2: If the current point $p_{i}=\left(x_{i},y_{i},z_{i}\right)$ is closer to the LiDAR than the previous point $p_{\left(i-1\right)}=\left(x_{\left(i-1\right)},y_{\left(i-1\right)},z_{\left(i-1\right)}\right)$, the current point $p_{i}$ is considered a special point.

(2) $$\sqrt{x_{i}^{2}+y_{i}^{2}}<\sqrt{x_{\left(i-1\right)}^{2}+y_{\left(i-1\right)}^{2}}$$

The points are sorted by radial distance magnitude, which may imply the presence of a small object if it is different from the sorting within the point set. As shown in Figure 3b, Rule 2 prevents blue points with low height values from being categorized as ground points, owing to their irregular cyclist profiles.

(2) Object point filtering rules

Considering that the points produced by LiDAR scanning objects are continuous and have certain height differences, we determine whether a special point is a small-object point by analyzing the height difference between the special point and a number of its previous points in the ordered point set.

Rule 1: Special points are categorized into three types according to the geometry of the small object: approaching, interior, and departure points. When a special point is detected, the height differences between the special point $p_{i}$ and the three points within the ordered point sets $p_{\left(i+1\right)}$ and $p_{\left(i+2\right)}$ is calculated, and if the height difference is positive and the absolute value exceeds the threshold Δh, the special point $p_{i}$ is categorized as an approaching point. After an approaching point is found, the next judgment condition is triggered to find a departing point, that is, the height difference between the special point $p_{j}$ and the two points within the ordered point set $p_{\left(j+1\right)}$ is calculated, and if the height difference is negative and the absolute value is lower than the threshold Δh, the special point $p_{j}$ is categorized as a departure point. Points between $p_{i}$ and $p_{j}$ in terms of radial distance are considered as object interior points. The calculation formula can be expressed as follows:

(3) $$\left\{\begin{array}{c} \left|z_{\left(i-1\right)}-z_{i}\right|>\Delta h,\;\;i=1,2\\ \left|z_{\left(j+1\right)}-z_{j}\right|<\Delta h,\;\;j=1\;\;\; \end{array}\right.$$

Rule 1 can distinguish small-object points from special points and avoid misclassifying the ground points in the special points as small-object points. As shown in Figure 3c, based on Rule 1, all point clouds of the cyclist can be categorized as object points.

Rule 2: In the point cloud data obtained from scanning, the point cloud adjacent to the LiDAR presents a higher density, resulting in smaller height differences between neighboring points; in contrast, the point cloud far away from the LiDAR presents a lower density, resulting in larger height differences between neighboring points. Therefore, it is inaccurate to use the same height threshold to classify all points. As a result, we use the adaptive elevation difference threshold, which is calculated as follows:

(4) Δh=ax+b

where x denotes the distance, and a and b are constants related to the LiDAR harness and mounting position, respectively. In order to determine the relationship between two points in the vertical direction of a point cloud as a function of distance, we used straight-line motion data of a reference object recorded by LIDAR from a real vehicle. We selected 150 frames of data from these and recorded the vertical distance between the points where the same two laser beams hit the reference object in each frame, as well as the distance of these points from the LiDAR sensor. We then curve-fitted these data to obtain their linear relationship using the linear model in Equation (4).

#### 3.1.2. Multi-Area Polar Coordinate Meshing

There are some limitations to the traditional polar coordinate meshing method. Grid redundancy occurs in the near region, which reduces the efficiency of ground segmentation. In the far region, the number of point clouds in the factorial grid is too small for ground feature values to be extracted. We adopted a multi-region polar coordinate meshing method to avoid these problems.

The point cloud space is divided into different regions $N_{i}$ based on the distance of the point cloud relative to the location of the LiDAR. For the mth region $N_{m}$ in the space, $p_{k}$ denotes the points in region $N_{m}$, and $\left(x_{k},y_{k}\right)$ are the projected coordinates of $p_{k}$, which have a Euclidean distance of $d_{k}=\sqrt{x_{k}^{2}+y_{k}^{2}}$ from the origin and an angle of $\theta_{k}=\tan^{-1}y_{k}/x_{k}$ with respect to the positive direction of the X axis of the LiDAR. In addition, the region with the maximum and minimum radial boundaries of $L_{max,m}$ and $L_{min,m}$, respectively, is divided into $N_{r,m}\times N_{\theta ,m}$ subgrids with different grid sizes for each region. Therefore, for each subgrid in region 14, the size is calculated as follows:

(5) $$\left\{\begin{array}{c} \frac{\left(i-1\right)\times \Delta L_{m}}{N_{r,m}}\leq d_{k}-L_{min},\;m<\frac{i\times \Delta L_{m}}{N_{r,m}}\\ \frac{\left(j-1\right)\times 2\pi }{N_{\theta ,m}}-\pi \leq \theta_{k}<\frac{j\times 2\pi }{N_{\theta ,m}}-\pi \end{array}\right.$$

where $\Delta L_{m}=L_{max,m}-L_{min,m}$ and $N_{\theta ,m}$ is related to the LiDAR parameter settings.

**Figure 4:**
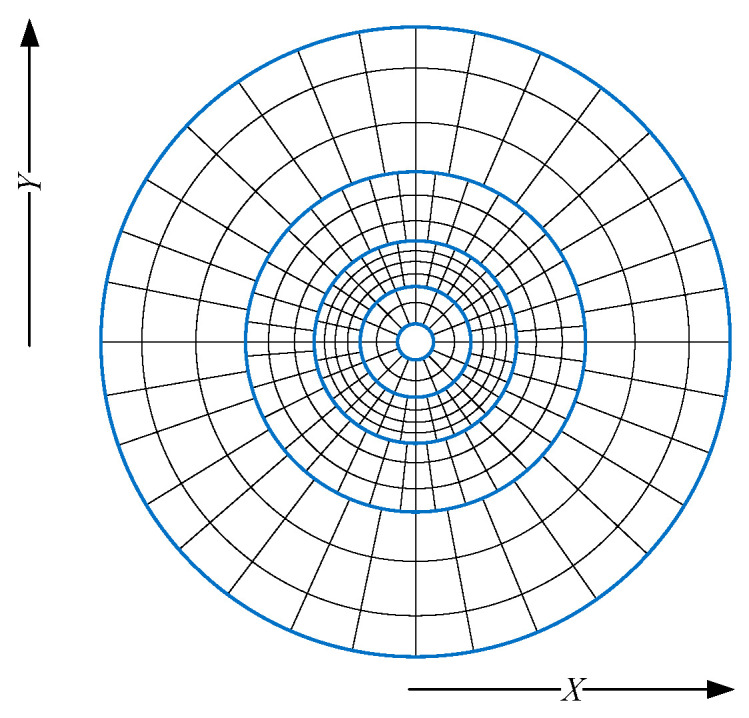
Multi-region polar gridding. The blue line is the area division line.

Considering the experimental environment, the total number of regions was set to four, as shown in Figure 4, for regions $N_{1}$ and $N_{4}$, which are the closest and farthest away from the LiDAR. Since LiDAR sensors emit laser beams at a constant angle, they generate denser point clouds when the laser hits nearby objects, while the point clouds become sparser at greater distances. The N4 region, being the furthest from the sensor, typically has the fewest point clouds. Additionally, the N1 region includes the blind spot of the LiDAR sensor, and some points in this region originate from the vehicle on which the sensor is mounted. These points are irrelevant to our detection task and are therefore removed in advance. As a result, the N1 region also has relatively fewer point clouds. A situation where the number of point clouds in the subgrid is too small leads to difficulty in ground feature extraction. To avoid this issue, the number of subgrids in the two regions is set to be small, and, while most of the point clouds are distributed in regions $N_{2}$ and $N_{3}$, the corresponding higher numbers of subgrids are set in regions $N_{2}$ and $N_{3}$ to enhance the ground feature extraction. This approach provides more subgrids to improve the accuracy of ground segmentation.

#### 3.1.3. Selection of Seed-Point Sets

The seed-point set, which is used to construct the initial planar model, must be selected at the end of the polar mesh segmentation. To better segment the pavement in different scenarios and speed up the iteration process, we use a double-thresholding method to select the ground seed-point set.

The point cloud data in a certain subgrid are denoted by $P_{i,j}=\left\{p_{1},p_{2},\ldots ,p_{n}\right\}$, and we calculate the average height threshold $h_{a\nu g}$ of all points in the subgrid using Equation (6) as follows:

(6) $$h_{a\nu g}=\overset{n}{\underset{i=1}{\sum }}\frac{p_{i}}{n}$$

If we rely only on the average height of all points within the subgrid $h_{avg}$ as the threshold, points with height values less than $h_{avg}$ are used as the initial seed-point set, and points greater than $h_{avg}$ are non-ground points, which may result in an unrealistic subgrid fit with all non-ground points. Although the process of selecting ground-fit points is iterative, incorrect selection of the initial seed-point set will lead to slower iterations. Therefore, a global threshold $H_{avg}$ is introduced to further constrain the process, and the average height of the point cloud in all regions is calculated as the global threshold using Equation (7):

(7) $$H_{avg}=\overset{m}{\underset{a=1}{\sum }}\frac{p_{a}}{m}$$

where $p_{a}$ denotes any point in all regions and m is the number of point clouds in all regions.

**Figure 5:**
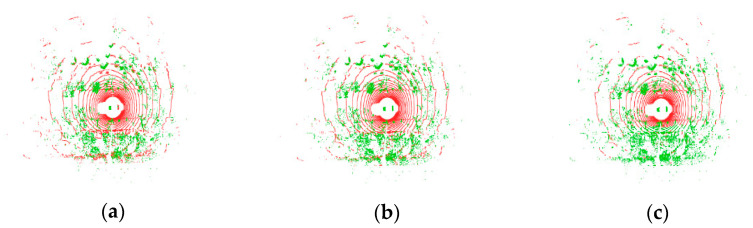
Seed-point set iteration process diagram. (**a**–**c**) depict the first, second, and nth iterations of the seed-point set, respectively. The red points are ground points.

The smaller of the two thresholds is selected as the final threshold of the subgrid, and all the points in the grid that are smaller than the final threshold are added to the initial seed-point set. The operation is performed again for the points in the initial seed-point set, which is continuously iterated to filter out the final seed-point set, as shown in Figure 5.

#### 3.1.4. Planar Model Fitting

**Figure 6:**
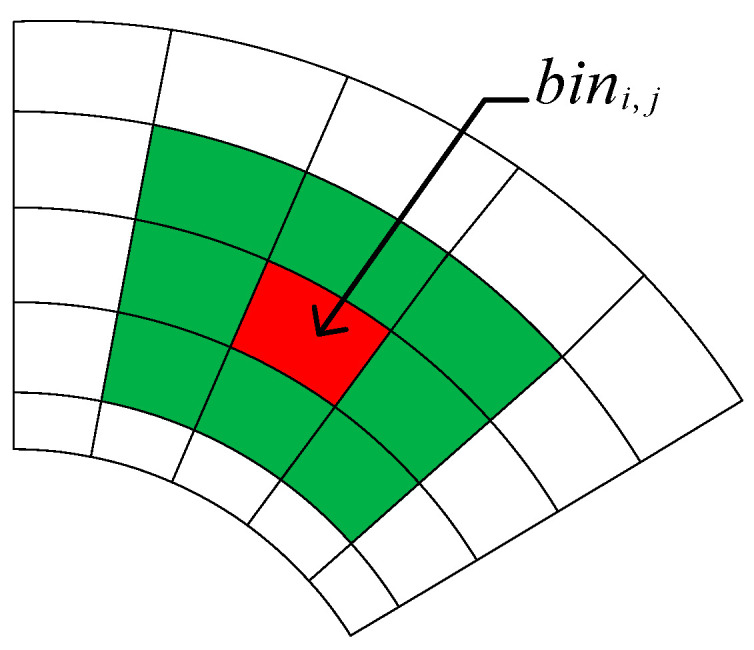
Schematic diagram of the reference normal vector, with the referenced subgrid in red and its surrounding neighborhood grid in green.

After the selection of the seed-point set, a planar model is required to describe the ground. We used a linear model for planar model estimation, by which the effective segmentation of ground points can be realized theoretically. However, in practical experiments, we found that planar point clouds such as streetlamps, walls, and car roofs in the interior of the subgrids are incorrectly recognized as ground points. To accurately fit the real ground, we set separate reference normal vectors for all subgrids. As shown in Figure 6, for any subgrid $bin_{i,j}$, its reference normal vector $\vec{n_{i,j}^{\prime }}$ is the average of the vector sum of the normal vectors of the surrounding neighborhoods.

The normal vector in the subgrid $bin_{i,j}$ is $\vec{n_{i,j}}$, and its angle $\nabla \theta_{i,j}$ with the reference normal vector $\vec{n_{i,j}^{\prime }}$ can be calculated by using Equation (8). By setting the clamp angle threshold ∇θ, the results obtained from plane-fitting in the region are judged; if $\nabla \theta_{i,j}$ is larger than the threshold ∇θ, it means that there are non-ground points in the three selected points $p_{1},$ $p_{2}$, and $p_{3}$, and it is necessary to re-select the three points to continue to fit until the eligible ground points are found for plane-fitting.

(8) $$\nabla \theta_{i,j}=\frac{\vec{n_{i,j}}\cdot \vec{n_{i,j}^{\prime }}}{\left|\vec{n_{i,j}}\right|\cdot \left|\vec{n_{i,j}^{\prime }}\right|}$$

After obtaining the fitted plane, to select ground points, it is also necessary to set a distance threshold D. The distances of the other points to plane $d_{i}$ are calculated using Equation (9). If $d_{i}<D$, the point is considered a ground point; otherwise, it is considered a non-ground point.

(9) $$d_{i}=\frac{\left|Ax+By+Cz+D\right|}{\sqrt{A^{2}+B^{2}+C^{2}}}$$

### 3.2. Small Objective Clustering Based on an Improved DBSCAN Algorithm

DBSCAN is a density-based clustering algorithm where data points within a region are connected under certain conditions to form a dense cluster. Before clustering begins, two parameters need to be set: the neighborhood radius (*Eps*) and the density threshold (*MinPts*), which significantly affect the clustering accuracy. The DBSCAN search process involves the following definitions.

(1) Neighborhood: For any point $p_{i}$, the circular region with $p_{i}$ as the center and the neighborhood radius (*Eps*) as the radius is called the neighborhood of $p_{i}$;
(2) Density Threshold (*MinPts*): The minimum number of points required in a neighborhood for a point $p_{i}$ to be considered a core point is called the *MinPts*;
(3) Core Point: For any point $p_{i}$, if the number of points in its neighborhood exceeds the *MinPts*, then $p_{i}$ is a core point;
(4) Border Point: For any point $p_{i}$, if the number of points in its neighborhood is less than the *MinPts*, then $p_{i}$ is a border point;
(5) Noise Point: If a point $p_{i}$ does not belong to any cluster, then $p_{i}$ is considered a noise point.

The basic principle of the DBSCAN algorithm is to select any point $p_{i}$ in the dataset as a starting point. If the number of points in the *Eps* neighborhood of $p_{i}$ is not less than the density threshold *MinPts*, then $p_{i}$ is a core point, and it, along with all the points in its neighborhood, is grouped into a cluster. The algorithm then continues by treating the neighborhood points as candidate points, repeating the process to search outward until no new points can be added. During this process, points that fall within the neighborhood of a core point but do not meet the core point criteria are classified as border points, and all remaining points are classified as noise points.

The traditional DBSCAN algorithm is prone to classifying the boundary point cloud of small objects as other clusters or noise if the parameters are not selected reasonably, and even classifying two small objects in one cluster. The traditional DBSCAN algorithm uses a fixed neighborhood-radius parameter. However, the distribution of LiDAR point clouds is denser near the sensor and sparser farther away. Using a fixed neighborhood-radius parameter cannot achieve ideal clustering results for the target point cloud. In addition, the determination of core points requires calculation of the distances between each point and all other points, which results in excessive computational effort. Therefore, we propose an improved DBSCAN algorithm, which is improved by optimization operations such as pre-clustering, setting an adaptive neighborhood radius, and reducing the number of traversed points; the specific steps are as follows:

(1) Before executing the DBSCAN algorithm, the k-means ++ algorithm is introduced to precluster the dataset. Any point from the point cloud data P is randomly selected as the initial cluster center $x_{1}$, and the probability $D_{i}\left(x\right)$ that the remaining data points $x_{i}$ become the cluster center is calculated according to Equation (10).
  (10) $$D_{i}\left(x\right)=\frac{d_{i}{\left(x\right)}^{2}}{\sum_{x_{i}\in P}d_{i}{\left(x\right)}^{2}}$$

In the formula, $d_{i}\left(x\right)$ represents the distance between point $x_{i}$ and the nearest cluster center. The point with the largest $D_{i}\left(x\right)$ is selected as the new cluster center.

The above steps are repeated to end the pre-clustering process by dividing the point cloud data into k groups using a lower number of iterations. The introduction of pre-clustering allows the point cloud data to be partitioned into k independent groups. By using the DBSCAN algorithm separately within different groups, the scope considered by the algorithm can be narrowed, and the clustering quality of the algorithm as well as its computational efficiency can be improved. The choice of k should be able to ensure that all point clouds are covered, while the number should be on the small side. If the k value is set as a large value, it will not only increase the iteration time of the algorithm, but also cause the point cloud to be overly segmented, resulting in the missed detection of the object. In this study, the k value of 5 is selected after several experimental tests in specific scenarios.

**Figure 7:**
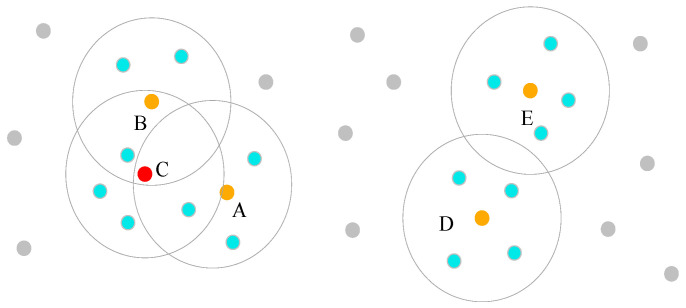
Improved way of searching for core points.

(2) Before performing refined clustering, the distribution characteristics of the point cloud that is dense nearby and sparse far away are used to select the appropriate neighborhood radius for data points with different distances. According to the relationship between the distance and neighborhood radius, the adaptive neighborhood radius is used, and its value can be calculated using Equation (11):
  (11) $$Eps=\frac{\lambda \pi \Delta \alpha D_{i}}{180^{{}^{\circ}}},$$
  where Δα is the horizontal angular resolution of the LiDAR, $D_{i}$ is the horizontal distance from the ith point to the LiDAR origin, and λ is the neighborhood radius coefficient. The value of λ depends on the relationship between neighboring point clouds and the distance of the LiDAR sensor, and is a fitting coefficient, which is affected by the actual parameters of the LiDAR sensor and the installation location. The horizontal angular resolution Δα of the LiDAR used in this paper is 0.16°, and λ takes the value of 1.3 after many scene-specific experimental tests.
(3) The main reason for the time-consuming nature of the traditional DBSCAN algorithm is that it must traverse all the data points; therefore, to improve the operational efficiency of the algorithm, we propose an improved method for searching for core points. First, any point in the point cloud data is selected as the starting point, the points exceeding the density threshold *MinPts* [18] are categorized as core points, and all the points in their neighborhood are removed. This step is repeated for the removed point cloud until all the core points are found. Finally, to avoid the existence of core points in the removed point cloud, the core points are considered as individual clusters, and intersecting clusters are merged. If there is a data point within the intersecting neighborhood space, the data point is categorized as a core point, and if there is a data point within the intersecting neighborhood space, the data point is categorized as a core point. If a data point exists within the intersecting neighborhood space, it is categorized as a core point and grouped into a cluster. As shown in Figure 7, because points A, B, D, and E satisfy the conditions of core points, they are judged as core points, but there point C is in the intersection region of the neighborhoods of points A and B. Point C is taken as a new core point, and because there is no data point in the intersection region of the neighborhoods of points D and E, the process of searching for core points is completed.
(4) The points in the neighborhood space of the core points are grouped into the same cluster as the core points to complete the intragroup clustering. To avoid the problem of the same cluster being split into two clusters based on grouping, the intergroup distance is first checked. If it is smaller than the specified threshold, then it indicates that there may be a clustering split; if the distance between the clusters is smaller than the distance threshold, then it indicates that the two clusters are in the same category, and the two clusters are combined into the same cluster. If the intergroup distance is larger than the specified threshold, then it indicates that there is no correlation between the two groups, and no further follow-up check is performed.

### 3.3. Construction of 3D Bounding Boxes

Object clustering classifies point clouds with similar features into the same cluster, and multiple clusters with different features are obtained at the end of clustering. To accurately describe the object position, size, and heading angle and to provide the necessary information for downstream tracking algorithms, it is necessary to enclose the point clouds of the same cluster using a 3D bounding box. We used the oriented bounding box (OBB) model [34] to extract small-object features and construct a bounding box.

## 4. Experiments and Results

### 4.1. Setup of Experimental Environment

**Experimental hardware environment construction**. The real-vehicle experimental platform used in our experiments was an intelligent vehicle based on the Haval H7. The platform was equipped with a Velodyne LiDAR unit, a ReelVision camera, inertial guidance–global positioning system (GPS) combined positioning, and a high-performance industrial computer.

A Velodyne HDL-32E LiDAR was used as the main sensor in the experiment to acquire the point cloud data of the surrounding environment of the intelligent vehicle. This LiDAR is a mechanically rotating LiDAR with the advantages of high resolution, high sampling rate, high stability, and easy integration, and is suitable for various autonomous driving tasks; its main parameters are listed in Table 1.

**Experimental software environment construction**. This experiment was performed on an Ubuntu 18.04 operating system based on the Linux kernel. This experiment focused on implementing the algorithm by writing code in Python 2.7 on a robot operating system (ROS). In the course of the experiment, the function of object-detection was realized by writing several ROS nodes and using the functional modules and libraries provided by the ROS to subscribe, process, and publish the point cloud data.

### 4.2. Ground-Segmentation Experiment and Results Analysis

**Data and evaluation indicators**. To fully evaluate the comprehensive performance of our proposed segmented ground-point cloud accurate segmentation algorithm, we collected 3000 frames of real campus road data through the real-vehicle experimental platform, during which the experimental vehicle was kept at an average speed of about 30 m/s. Because the ground type and ground object were similar in neighboring frames, 300 frames of noncontinuous and representative sample data were selected for the experiment. To accurately analyze the ground-segmentation effect, we used the open-source 3D point cloud processing software Cloud Compare V2 to manually label the ground and non-ground points as real values to measure the segmentation results of the algorithm. 

In this experiment, the precision rate P and recall rate R evaluation metrics proposed by Shengming et al. [35] were used to quantitatively evaluate the effectiveness of the ground-segmentation algorithm.

After repeated experiments in the preliminary stage, the thresholds used in our ground-segmentation algorithm were determined. The specific parameters are listed in Table 2, where $N_{max}$ is the maximum number of iterations of the ground-segmentation algorithm.

**Analysis of ground-segmentation results**. Intelligent vehicles interact with cyclists and pedestrians in many scenarios. We selected the rough road-surface and sloped road-surface scenarios to qualitatively evaluate our proposed segmentation algorithm and compare the segmentation results with random sample consensus (RANSAC) [36] and R-GPF [25].

The rough surface scenario was selected to test the algorithm’s ability to handle complex textures and uneven terrain. Such surfaces are common in real-world environments, and accurately processing data under these conditions is critical for the algorithm’s practical application. The sloped surface scenario was chosen to evaluate the algorithm’s performance in detecting and processing inclined planes, which pose unique challenges due to changes in the height and angle of view. The variability introduced by roughness and slope tests the algorithm’s adaptability to different road conditions. 

**Figure 8:**
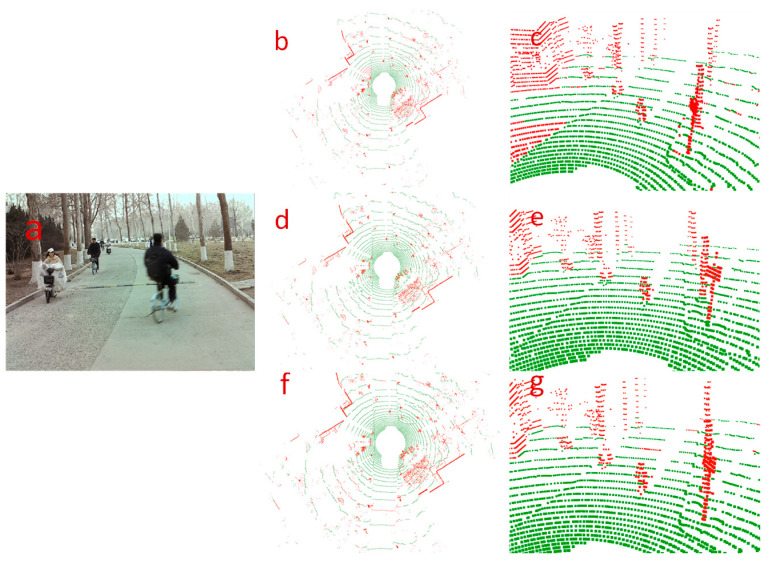
Comparison of segmentation results in the rough road scene. The first row corresponds to the results processed by the RANSAC algorithm, the second row corresponds to the results of the R-GPF algorithm, and the third row corresponds to the results of our proposed ground-segmentation algorithm. The left side (**a**) is the scene map captured by the front-view camera, the center position (**b**,**d**,**f**) is the global ground-segmentation effect map of each algorithm, and the right side (**c**,**e**,**g**) is the local ground-segmentation effect map of each algorithm, in which the green dots are the ground dots and the red dots are the non-ground dots.

In Figure 8, we show a qualitative comparison of the segmentation effect of our algorithm with two control algorithms in a rough road-surface scene. In the scene graph, there is a rough road-surface on the left side of the road in the current frame, along with obstacles such as bicyclists and street trees. By comparing the segmentation effects, it was seen that the control algorithms recognized some object points as ground points, as shown in Figure 8c,e. Meanwhile, it can be seen from Figure 8c that RANSAC also incorrectly recognized the rough ground-point cloud as object points. Our algorithm more accurately segmented the ground and correctly recognized all point clouds of the cyclist.

**Figure 9:**
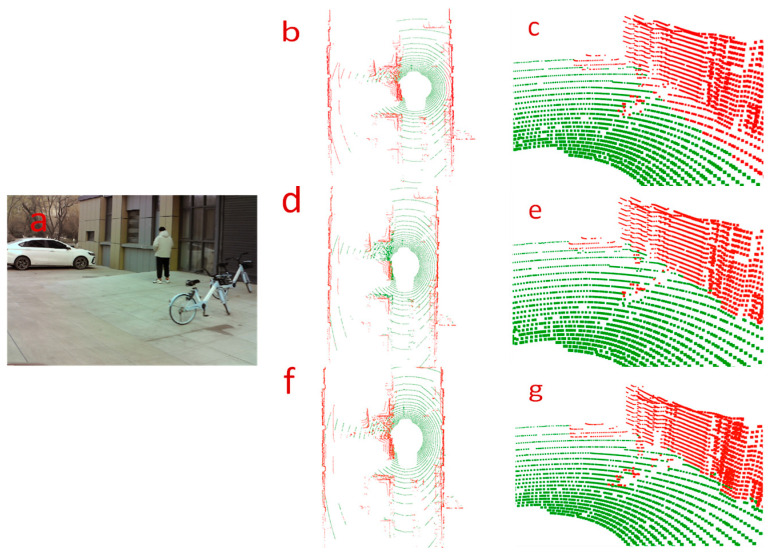
Comparison of segmentation results for the sloped pavement scene. The first row corresponds to the results processed by the RANSAC algorithm, the second row corresponds to the results of the R-GPF algorithm, and the third row corresponds to the results of our proposed ground-segmentation algorithm. The left side (**a**) is the scene map captured by the front-view camera, the center position (**b**,**d**,**f**) is the global ground-segmentation effect map of each algorithm, and the right side (**c**,**e**,**g**) is the local ground-segmentation effect map of each algorithm, in which the green dots are the ground dots and the red dots are the non-ground dots.

Figure 9 shows a qualitative comparison of the segmentation results of our algorithm and the two control algorithms for a sloping road-surface scene. From the scene graph, we can see that there is sloping ground in the current frame, and there are pedestrians and parked bicycles on the slope. By comparing the global ground-segmentation effect graphs of each algorithm, it can be seen that the three algorithms realize the segmentation of the ground and obstacles. Comparing the local segmentation graphs, it can be observed that both control algorithms misidentified small-object points as ground points, as shown in Figure 9c,e, whereas the RANSAC algorithm performed poorly in segmenting the ground on the slope, as shown in Figure 9c. In Figure 9f,g, it can be seen that our proposed ground-segmentation algorithm is not only able to accurately segment flat and sloping ground but also correctly recognize the points with lower height values among pedestrians and bicycles as non-ground points.

According to the above analysis, the segmentation effect of our algorithm is better, and it can effectively and robustly segment the ground-point cloud and small-object point clouds in different road scenes.

To quantitatively analyze the accuracy and real-time performance of the algorithm, we processed 300 frames of sample data using the above three algorithms and calculated the precision rate (P), recall rate (R), accuracy rate (A), F1 score (F1), average running time used to process one frame of sample data, and frames per second (FPS) of the three algorithms. 

The calculation formulas for the precision rate (P), recall rate (R), accuracy rate (A), and F1 score (F1) are as follows:

(12) $$P=\frac{S_{NTP}}{S_{NTP}+S_{NFP}}\;R=\frac{S_{NTP}}{S_{NTP}+S_{NFN}}$$

(13) $$A=\frac{S_{NTP}+S_{NTN}}{S_{NTP}+S_{NTN}+S_{NFP}+S_{NFN}}\;F1=\frac{2\times S_{NTP}}{2\times S_{NTP}+S_{NFP}+S_{NFN}}$$

In the formula, $S_{NTP}$ represents the true positives for ground segmentation, meaning the number of points correctly classified as ground points. $S_{NFP}$ represents the false positives, meaning the number of points incorrectly classified as ground points. $S_{NTN}$ represents the true negatives, meaning the number of points correctly classified as non-ground points. $S_{NFN}$ represents the false negatives, meaning the number of points incorrectly classified as non-ground points. The results are shown in Table 3.

As can be seen in Table 3, our algorithm outperforms the two control algorithms in terms of precision, accuracy, F1 score, and recall in different road-surface scenarios, while meeting real-time requirements. This proves the performance advantage of our algorithm. Among the algorithms, the recall rate of the RANSAC algorithm is only 86.52%, which is lower than those of the R-GPF algorithm and our algorithm. This is because the RANSAC algorithm has more stringent constraints in fitting planes, and it is more inclined to incorrectly recognize ground points as non-ground points. R-GPF has the shortest average runtime for processing a frame of point cloud data and has the advantage of a high recall rate, but its precision rate is lower, which is because the algorithm recognizes too many non-ground points as ground points. Our algorithm uses the small-object point cloud distribution characteristics to coarsely filter the point cloud data, which makes the algorithm’s precision and recall higher. However, the amount of data processed increases simultaneously, resulting in a slightly lower running speed than R-GPF, and this slightly reduces the FPS, but 30.8 FPS is still above the industry standard of 20 FPS for real-time performance and therefore still meets the real-time requirements.

### 4.3. Cluster Detection Algorithm Experiments and Results Analysis

**Data and evaluation indicators**. To verify the clustering effect of the proposed improved DBSCAN algorithm on small-object point clouds in real scenes, we first used the real-vehicle experimental platform to collect and select 300 frames of point cloud data in different scenes as samples, and then used our proposed ground-segmentation algorithm to process the point cloud data in the samples. Finally, we used the traditional DBSCAN and the improved DBSCAN algorithms to cluster the processed point cloud data, and constructed the enclosing frame through the OBB model to compare the two clustering detection effects.

**Figure 10:**
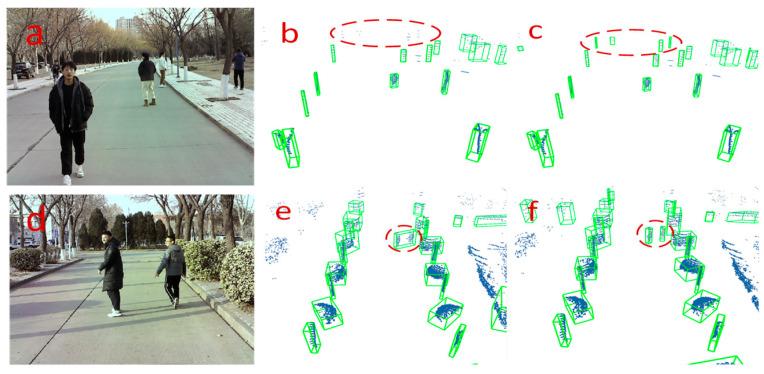
Comparison of the visualization results of the two algorithms for clustering detection. The left side (**a**,**d**) shows different scene maps captured by the camera, the middle (**b**,**e**) shows the clustering detection results of the traditional DBSCAN algorithm, and the right side (**c**,**f**) shows the clustering detection results of our algorithm. The blue points in the detection map are point clouds, and the green box is the bounding box fitted by the algorithm.

**Analysis of the results of the small-object clustering experiment**. We present a qualitative comparison of the clustering effects of our clustering algorithm and the DBSCAN algorithm in Figure 10, where there are street trees, cyclists, and pedestrians at different distances from the experimental car in the scene. In the first frame of the point cloud, the traditional DBSCAN algorithm fails to cluster the pedestrians, cyclists, and street trees within the red elliptical box at a significant distance (Figure 10b), whereas our algorithm succeeds in clustering and detecting all four objects (Figure 10c). In the second frame of the point cloud, the traditional DBSCAN algorithm clusters two pedestrians in a red elliptical box to detect one obstacle (Figure 10e), whereas the improved algorithm correctly segments and detects two pedestrian objects (Figure 10f). In summary, our algorithm can effectively resolve the difficulty of distinguishing neighboring small objects and reduce the instances of missing small objects at a distance.

To verify the accuracy and real-time performance of our improved DBSCAN algorithm for cluster detection, we counted the number of pedestrians, cyclists, and vehicles in the 300 frame samples, which was 321, 337, and 327, respectively, and recorded the number of correct cluster detections and processing time of the DBSCAN algorithm before and after the improvement. The recall of the clustering detection, that is, the ratio of the number of correct detections to the total number of objects, was used as an evaluation metric for the accuracy of the algorithms; the real-time performance of the algorithms was evaluated using the average time used for clustering one frame of the point cloud and the FPS. The clustering detection results of the two algorithms are listed in Table 4.

As can be seen from Table 4, the improved DBSCAN algorithm increases the number of correct detections by 93 compared to the traditional algorithm, and the recall rate is increased by 9.44%, whereas the improved core-point search method reduces the average running time by 7.96 ms compared with the traditional algorithm. The experimental results verify that our improved DBSCAN algorithm better clusters the objects for detection in terms of accuracy and real-time performance.

**Figure 11:**
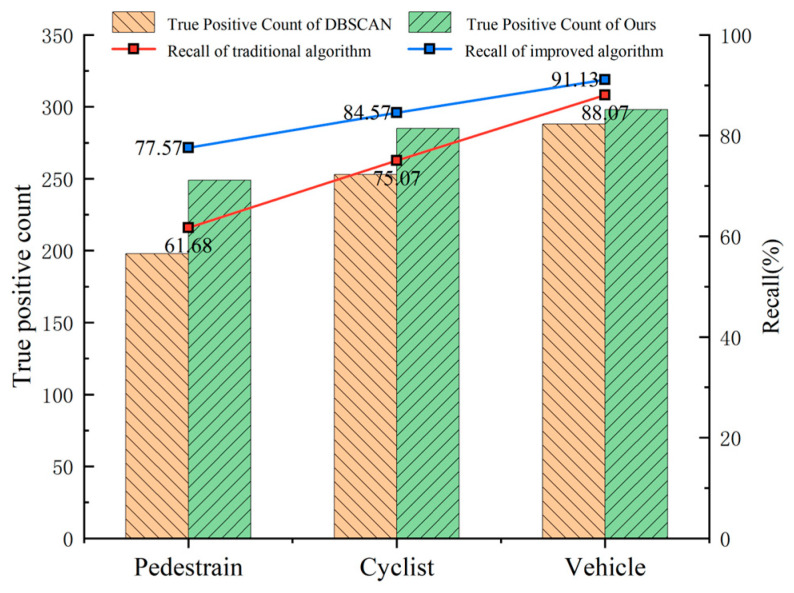
Comparison of two algorithms for different types of clustering detection results.

In addition, to verify the clustering detection effect of our algorithm on small objects, we counted the number of correctly detected pedestrians, cyclists, and vehicles and calculated the recall rate; the results are shown in Figure 11.

Figure 11 shows that our improved DBSCAN algorithm is better than the traditional DBSCAN algorithm in detecting all three objects, and the recall of the pedestrian and cyclist categories is improved by 15.89% and 9.50%, respectively, proving the feasibility of detecting small objects by clustering our proposed improved DBSCAN algorithm.

## 5. Discussion and Conclusions

In this study, we propose an object-detection algorithm based on point-cloud clustering. A segmented ground-point cloud accurate segmentation algorithm is proposed which applies heuristic rules to the coarse filtering of small-object point clouds, as well as fine filtering by a ground-segmentation method based on plane-fitting. We experimentally proved that our proposed ground-segmentation method can robustly and accurately segment ground-point clouds that are difficult to segment in different scenarios. We also propose an improved DBSCAN clustering algorithm that enhances the clustering accuracy by pre-clustering and setting the adaptive neighborhood radius, which improves the algorithm running speed by enhancing the core point search method, and is better than the original algorithm in terms of its accuracy rate. Our improved algorithm is also able to resolve the under-segmentation of neighboring objects and the failure to efficiently detect distant small objects.

In addition, we used visualization experiments to prove that our proposed small-object-detection algorithm, based on the point-cloud clustering method, can realize the accurate detection of small objects.

The limitation of our object-detection algorithm based on point-cloud clustering is that it is less effective in detecting more complex scenes (e.g., lively downtown areas and intersections). Therefore, we expect that future work will adapt to more complex traffic environments and further improve the accuracy of object detection.

## Figures and Tables

**Table 1:** Main parameters of the Velodyne HDL-32E LiDAR.

Parameters	Values
Laser Harness (Wire)	32
Measuring range (m)	80–100
Accuracy (cm)	±2
Field of view (°)	360 × 41.34
Supply Voltage (VDC)	9–32
Power (W)	31.4
Output (points per second)	700,000

**Table 2:** Ground segmentation experimental parameters.

Parameter	Value	Parameter	Value
$\alpha_{\mathrm{th}}$	20°	∇θ	3.3°
a	0.025	D	0.2 m
b	−0.05	$N_{max}$	5

**Table 3:** Comparison of different ground-segmentation algorithm results.

Algorithms	Precision Rate P	Recall Rate R	Accuracy Rate A	F1 Score F1	Average Running Time	FPS
RANSAC	91.66%	86.52%	85.82%	89.02%	69.52 ms	14.4
R-GPF	89.26%	91.33%	89.27%	90.29%	28.55 ms	35.0
Ours	91.86%	92.70%	90.94%	92.28%	32.47 ms	30.8

**Table 4:** Comparison of clustering performance of the DBSCAN algorithms before and after improvement.

Clustering Algorithm	Object Number	Number of Correct Detections	Recall Rate	Average Running Time	FPS
DBSCAN	985	739	75.03%	57.85 ms	17.3
Ours	985	832	94.47%	49.89 ms	20.0

## Data Availability

The authors declare that upon reasonable request, the data and the code are available from the corresponding author.
